# Mechanotransduction: from the cell surface to the nucleus via RhoA

**DOI:** 10.1098/rstb.2018.0229

**Published:** 2019-07-01

**Authors:** Keith Burridge, Elizabeth Monaghan-Benson, David M. Graham

**Affiliations:** Department of Cell Biology and Physiology, and Lineberger Comprehensive Cancer Center, University of North Carolina, Chapel Hill, NC 27599, USA

**Keywords:** RhoA, mechanotransduction, nucleus, cell adhesion molecules, cytoskeleton, fibrosis

## Abstract

Cells respond and adapt to their physical environments and to the mechanical forces that they experience. The translation of physical forces into biochemical signalling pathways is known as mechanotransduction. In this review, we focus on two aspects of mechanotransduction. First, we consider how forces exerted on cell adhesion molecules at the cell surface regulate the RhoA signalling pathway by controlling the activities of guanine nucleotide exchange factors (GEFs) and GTPase activating proteins (GAPs). In the second part of the review, we discuss how the nucleus contributes to mechanotransduction as a physical structure connected to the cytoskeleton. We focus on recent studies that have either severed the connections between the nucleus and the cytoskeleton, or that have entirely removed the nucleus from cells. These actions reduce the levels of active RhoA, thereby altering the mechanical properties of cells and decreasing their ability to generate tension and respond to external mechanical forces.

This article is part of a discussion meeting issue ‘Forces in cancer: interdisciplinary approaches in tumour mechanobiology’.

## 1. Introduction

Cells are continuously subjected to a wide variety of mechanical forces. This is well illustrated by tumour cells, which experience many different forces as tumours grow and invade. As a solid tumour grows, it typically becomes denser and the tumour cells will be exposed to a more rigid physical environment. Some of this is due to the deposition of more extracellular matrix (ECM) but it also arises both from cell proliferation leading to tighter packing of cells within the available tissue space and increased contractility of stromal cells [1–4]. Various forces are encountered by tumour cells that are metastasizing, whether this occurs by single cell invasion or by collective cell migration [5]. Squeezing through confined spaces in the ECM exposes tumour cells to significant compressive and tensile forces, which can be sufficient to induce transient rupture of the cells' nuclei, release of chromatin and induce DNA damage [6,7]. Those tumour cells that metastasize to distant sites typically pass into lymphatics or blood vessels by intravasation. This involves a series of forces as the invading cells pass through the subendothelial basement membrane and then between the endothelial cells lining the vessel. Transport in the blood circulation will expose tumour cells to an extremely different mechanical environment, where the cells experience shear stress from fluid flow, as well as repeated collisions, both with other circulating cells and with the vessel walls. At distant sites, the tumour cells need to extravasate out of the circulation and again this will involve a series of different mechanical forces as the cells cross the vessel wall and underlying matrix to enter the target tissue. Typically, this can be expected to have a different mechanical environment from the tissue of tumour origin.

While it has long been recognized that chemical signals regulate cell behaviour, it is now well accepted that mechanical forces also play a critical role in regulating cellular function. Many signalling pathways are activated in response to different forms of mechanical force being exerted on the cell surface. These range from stretch-activated ion channels to activation of kinase cascades and Rho GTPases [8,9]. Ultimately, many of these pathways affect gene transcription. Space limitations prevent us from considering many of these topics and here we will focus on two: how mechanical tension exerted on cell adhesion molecules affects the RhoA signalling pathway, and secondly, the role of the nucleus as a physical structure in mechanotransduction signalling.

## 2. RhoA signalling in mechanotransduction

**Figure 1.**
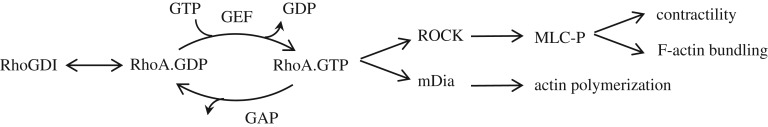
RhoA regulation. RhoA bound to GDP is inactive and can be sequestered by Rho guanine nucleotide dissociation inhibitor (RhoGDI). GDP exchange for GTP is promoted by guanine nucleotide exchange factors (GEFs), whereas GTP hydrolysis is stimulated by GTPase activating proteins (GAPs). In the active, GTP-bound state, RhoA activates the kinase ROCK which promotes contractility and bundling of actin filaments by activating myosin via phosphorylation of the regulatory myosin light chain (MLC). RhoA also binds and activates the formin mDia, stimulating actin polymerization.

The RhoA signalling pathway is central to mechanotransduction because it plays a key role in regulating the actin cytoskeleton and its response to mechanical force [10]. RhoA belongs to the Rho family of small GTPases, for which the mammalian genome encodes approximately 20 members [11]. Rho GTPases act as molecular switches, cycling between an active GTP-bound state and an inactive GDP-bound state. The activity of Rho GTPases is regulated by three classes of proteins: Guanine nucleotide Exchange Factors (GEFs), GTPase-Activating Proteins (GAPs) and Guanine nucleotide Dissociation Inhibitors (GDIs) (figure 1). GEFs activate Rho proteins by catalysing the exchange of GDP for GTP, whereas GAPs promote the intrinsic GTPase activity leading to the hydrolysis of GTP to GDP and inactivation [12,13]. GDIs extract membrane bound GTPases into the cytosol, where they are sequestered in their inactive conformation [14]. Rho proteins regulate a wide variety of mechanically sensitive cellular functions including cytoskeletal organization, cell polarity, proliferation and differentiation [15].

Contractile forces are largely generated by the interaction of myosin II with actin filaments. GTP-bound RhoA regulates myosin II activity by stimulating Rho kinase (ROCK), which, in turn, enhances the phosphorylation of the regulatory myosin light chain (MLC). This is accomplished both by direct phosphorylation of the regulatory MLC [16] and through the phosphorylation and consequent inhibition of the MLC phosphatase (MYPT) [17]. MLC phosphorylation enhances the assembly of myosin II into filaments and promotes its ATPase activity, thereby increasing the contractile force exerted by myosin II on actin filaments. Assembled into filaments myosin is also a very effective bundler of filamentous actin. Additionally, ROCK phosphorylates and activates LIM kinase allowing it to phosphorylate and inhibit the actin-severing protein cofilin [18]. This enhances actin filament stability. RhoA also stimulates further actin filament assembly through its effector mDia, an actin nucleating protein in the formin family [19]. Consequently, RhoA signalling is largely responsible for much of the intracellular force generation within cells [20].

## 3. Mechanical force and RhoA guanine nucleotide exchange factor activation

**Figure 2.**
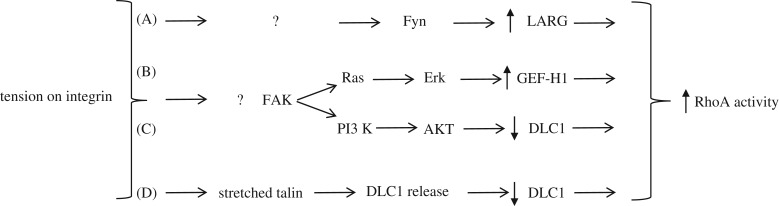
Pathways by which tension on integrins may increase RhoA activity. In (A) the tyrosine kinase Fyn is activated in an unresolved manner. It phosphorylates and activates the Rho GEF LARG. In (B), FAK is activated and via the Ras/Erk pathway activates GEF-H1. In (C), downstream from FAK, PI3 kinase (PI3 K) is activated triggering the activation of AKT, which phosphorylates the Rho GAP DLC1, thereby decreasing its activity. In (D), DLC1 is active when bound to talin but tension transmitted from integrins to talin, stretches talin, releasing DLC1 allowing it to adopt an inactive conformation. Some of these pathways may act in parallel and synergize to stimulate RhoA activity in response to tension exerted on integrins.

A role for RhoA was established in mechanotransduction downstream from tension exerted on fibronectin-coated beads adhering to the cell surface [21] and subsequent work showed that tension on integrins activates RhoA [22]. For activation to occur, this requires either activation of a GEF or inhibition of a GAP. Identifying which ones may be involved has been a challenge because the number of GEFs and GAPs greatly exceeds the number of Rho GTPases, with some showing limited specificity but others acting on many different family members [23]. Using a strategy to identify active GEFs, both GEF-H1 and LARG were identified, being activated in response to tension on integrins [24]. Exploring signalling upstream of these GEFs, LARG was found to be activated as a result of phosphorylation by the Src family kinase Fyn, whereas GEF-H1 was activated by the MEK/ERK pathway downstream of FAK activation (figure 2) [24]. Somewhat similar results were observed by another group who used the same approach of pulling on fibronectin-coated beads, but in these experiments the beads were adhered to endothelial cells rather than fibroblasts [25]. These investigators found that GEF-H1 and p115RhoGEF were activated; however, LARG was not. It should be noted that LARG and p115RhoGEF are very similar and belong to the same subfamily of GEFs. Exploring the pathway leading to activation, FAK was again implicated and, additionally, a role for one of the isoforms of Shc coupling to FAK was demonstrated to be critical.

The activation of GEFs in response to mechanical force on fibronectin-coated beads is a downstream response to tension-mediated activation of kinases, particularly members of the Src family of kinases (SFKs) and/or FAK. SFKs become activated very rapidly following mechanical tension [26]. How might these kinases be activated? Several pathways have been suggested. One involves protein tyrosine phosphatases (PTPs) associated with integrins, such as RPTPα [27]. Evidence has been presented that RPTPα forms a functional complex with the integrin αVβ3 and that in response to mechanical tension RPTPα activates SFKs by removing the inhibitory phosphorylation from the tail of SFKs that, when present, maintains them in an inactive form [27]. Alternatively, some integrin β-chain cytoplasmic domains have been shown to bind to SFK SH3 domains [28], although whether this specifically activates SFKs in response to tension has not been established. In many situations there is a close relationship between the activation of SFKs and FAK [29,30]. Many studies have shown that FAK is activated downstream from integrin engagement and formation of focal adhesions [31,32]. Furthermore, inhibiting tension on integrins by inhibiting myosin activity with blebbistatin decreases FAK activation [33]. Tension on integrins was shown to promote integrin engagement with the synergy site on fibronectin beyond the RGD binding site and that this additional engagement promoted the activation of FAK [34]. Consistent with this finding, others have shown that tension on fibronectin exposes the synergy binding site such that the integrin α5β1 binds to this as well as to the RGD binding site [35]. In this latter study, it was argued that FAK becomes clustered as a result of full integrin–fibronectin engagement and that clustered FAK trans-phosphorylates leading to FAK activation. The activated state of FAK is sustained by the FAK FERM domain binding to PIP2 maintaining FAK in the open active conformation [35]. Using molecular dynamics and mechano-biochemical simulations it has been argued that FAK can be opened up and activated as a direct result of mechanical tension. This model derives from the N-terminal FERM domain of FAK being able to bind to PIP2 in the membrane and the C-terminal focal adhesion targeting domain of FAK interacting with the cytoskeleton. As a result, tension can be exerted across FAK to release it from the auto-inhibited conformation [36]. Other proteins such as talin are stretched in response to mechanical tension exerted on integrin-mediated adhesions [37]. It will be interesting to learn whether these other deformations occur in parallel with or whether there is a sequential stretching and activation of proteins such as talin, Src, FAK, etc.

Using magnets to apply force on magnetic beads coated with antibodies has allowed several different adhesion molecules to be examined for how they influence RhoA activity. Interestingly, most reveal RhoA activation but sometimes the same GEFs are involved and sometimes different ones. Thus, applying force on the endothelial adhesion molecule PECAM activated GEF-H1 and LARG [38], whereas ICAM-1, also in endothelial cells, activated LARG but not GEF-H1 [39]. Tension on JAM-A in endothelial cells activated GEF-H1 and p115RhoGEF, but not LARG [40]. A pattern emerges that applying tension on a variety of cell adhesion molecules activates one or two of a group of GEFs that includes GEF-H1 and the closely related GEFs, LARG and p115RhoGEF. Whether the differences reflect different adhesion molecule complexes, or differences between cell types and pulling regimes, has not been resolved.

Mechanical force can be exerted experimentally on cells in a variety of ways. Many studies have examined cell behaviour in response to cyclic stretching of cells plated on deformable substrata. Using this approach with mesangial cells of the kidney, RhoA activation was shown to occur in response to the GEF Vav2, which was tyrosine phosphorylated and activated by Src [41]. However, cyclic stretching of pulmonary endothelial cells resulted in activation of GEF-H1, which was found to be dependent on stretch-induced microtubule disassembly [42]. GEF-H1 was also found to be the relevant GEF in cells responding to rigid substrata [43]. In this situation, tension is generated internally by myosin II and this tension is exerted on the cells' focal adhesions.

## 4. Mechanical force and Rho GTPase-activating proteins

In response to integrin engagement with the ECM, there is an initial depression of RhoA activity [44], caused by Src-mediated activation of p190RhoGAP [45]. A similar depression in RhoA activity was observed in endothelial cells responding to fluid shear stress [46], and again the decrease in RhoA activity was shown to be due to p190RhoGAP [47]. This is a situation where mechanical force (shear stress) activates a Rho GAP, but can force-mediated *inhibition* of Rho GAPs also contribute to RhoA activation? Recent evidence strongly supports this idea. Two Rho GAPs have particularly caught the attention of those interested in mechanotransduction and RhoA activity: the tumour suppressor Deleted in liver cancer 1 (DLC1) and p190RhoGAP. Recently, DLC1 was shown to be inhibited by AKT phosphorylation downstream from receptor tyrosine kinase activation and this elevated RhoA activity in response to insulin, EGF and insulin-like growth factor [48]. Since AKT is also activated downstream of mechanical force applied to integrins [49], it seems likely that the inhibition of DLC1 by this pathway may also contribute to elevated RhoA activity in response to mechanical stress (figure 2).

DLC1 is notable because it is recruited to focal adhesions [50]. One of its binding partners in focal adhesions is the mechanosensitive protein talin. DLC1 binds to the R8 domain of talin, which both localizes and activates DLC1 to decrease active RhoA levels. However, upon tension sufficient to stretch talin and open up the R8 domain, DLC1 is released in a conformationally inhibited form, contributing to increased RhoA activity [51]. The release and consequent inactivation of DLC1 from stretched talin points to another way that mechanical tension exerted on integrin adhesions may increase RhoA activity (figure 2). This work, however, also suggests a potential negative feedback pathway which may be important in limiting the size of focal adhesions. It has been known for some time that mechanical tension promotes the growth of focal adhesions in a RhoA-dependent manner [52]. Tension on focal adhesions stretches components such as talin to recruit additional binding partners [37]. However, large adhesions have been determined to generate less traction force than small adhesions [53], and FRET-based tension sensors have revealed that less tension is transmitted across components in large rather than small focal adhesions [54–56]. A negative feedback loop must be operating to prevent the continued growth of focal adhesions in response to increasing tension. One possible pathway is suggested by the recent work on DLC1 [51]. As focal adhesions grow in response to tension, the recruitment of more talin will result in the tension across the adhesion being carried by more talin molecules such that individual talin molecules each bear less load. This decreased tension on talin will allow the R8 domain to refold and consequently to sequester and activate DLC1. In turn, this should decrease RhoA activity and decrease the tension being generated and exerted on the larger focal adhesions. Consequently, the growth of focal adhesions is predicted to cease. Undoubtedly, other mechanisms also counteract the continued growth of focal adhesions driven by tension. This is an interesting topic that merits further investigation. It will probably involve negative feedback pathways inhibiting GEFs and activating GAPs to decrease RhoA activity. Additionally, other mechanisms likely come into play, such as tension-stimulated internalization of integrins and other disassembly mechanisms.

**Figure 3.**
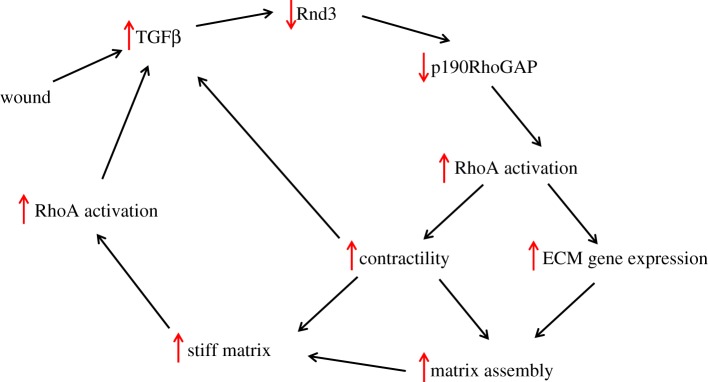
RhoA signalling in fibrosis. A positive feed-forward cycle is illustrated. Transforming growth factor-beta (TGFβ) is released in response to tissue wounding. TGFβ represses synthesis of Rnd3 causing a decrease in p190RhoGAP activity and a consequent increase in RhoA activity. The increase in active RhoA stimulates both myosin-mediated contractility and increased expression of ECM genes. In turn, these both promote increased matrix assembly resulting in a stiffer matrix. The stiff matrix further stimulates elevated RhoA activity. The high levels of contractility and the stiff matrix promote release of more active TGFβ from its inactive matrix-bound state, continuing the cycle.

The inhibition of p190RhoGAP has been identified as another cause of elevated RhoA activity in situations of mechanical tension. This was discovered in studies of fibroblasts from patients with idiopathic pulmonary fibrosis (IPF) [57]. The causes of IPF are not clear but elevated levels of TGFβ are a major factor promoting fibrosis and there is much evidence indicating a major role for RhoA in this disease, as well as other types of fibrosis. Exploring the signalling pathways that elevate RhoA activity, it was found that p190RhoGAP activity was depressed both in fibrotic fibroblasts and in response to TGFβ [57]. Investigating the mechanism revealed that expression of Rnd3/RhoE, an activator of p190RhoGAP, was suppressed by TGFβ. A characteristic of fibrotic tissues is their increased stiffness which arises from the deposition of excess ECM. Elevated stiffness enhances RhoA activity [58] and GEF-H1 has been implicated [43]. Notably, Rnd3 expression and p190RhoGAP activity are also both decreased in fibroblasts adhering to rigid substrata, suggesting that this pathway also contributes to elevated RhoA activity in cells exposed to rigid environments [57]. Because TGFβ activity is also stimulated by increased mechanical tension and growth on rigid substrata [59], this suggests a positive feed-forward pathway involving Rnd3 and p190RhoGAP, which is illustrated in figure 3. It should be noted that because Rnd3 expression is regulated transcriptionally, this provides a relatively slow mode of regulation of RhoA activity compared with the regulation that is mediated by phosphorylation of GEFs or GAPs.

Fibrosis and increased stiffness are characteristics of many solid tumours and promote tumour growth. Culturing breast epithelial cells on soft versus rigid collagen gels reveals that as rigidity increases there is decreased epithelial tubulogenesis, increased contractility and enhanced proliferation [3,58,60]. Growth in a more rigid environment was also shown to promote an invasive phenotype [61]. Underlying many of these characteristics is the enhanced activity of RhoA in cells growing within a rigid environment. In preliminary experiments we have found that Rnd3 levels are depressed when normal and breast cancer epithelial cells are cultured on more rigid substrates (E Monaghan-Benson 2018, unpublished results), suggesting that this pathway may be important in elevating RhoA activity in tumours. In previous work we found that drugs such as nintedanib and pirfenidone, which have been used to treat IPF [62,63], elevate Rnd3 expression and thereby decrease RhoA activity [57]. This raises the possibility that these drugs may also have therapeutic potential for solid tumours.

## 5. Role of the nucleus in mechanotransduction

Much evidence supports bidirectional mechanical signalling between the nucleus and the cytoskeleton. The mechanical properties of the nucleus have been of interest for some time and were particularly stimulated when mutations in Lamin A/C were found to underlie a range of genetic diseases with a mechanochemical basis [64]. These laminopathies include forms of Emery–Dreifuss muscular dystrophy, limb girdle muscular dystrophy and dilated cardiomyopathy, but most strikingly Hutchinson–Gilford Progeria [65]. In this premature ageing disease the nuclei reveal altered shape, often being lobulated, and have a thickened nuclear lamina and loss of peripheral heterochromatin [66]. The nuclei are stiffer, and the cells display altered mechanical properties having more apoptosis in response to mechanical strain [67,68].

The idea that the nucleus is mechanically connected to the cytoskeleton goes back a long way [69]. Penman's group showed electron micrographs of cytoskeletal elements enveloping and appearing to connect with the outer nuclear membrane [70]. Later it was shown that applying tension at the cell surface causes distortion of the nucleus, confirming that tension is transmitted to this organelle via the cytoskeleton [71]. Nuclear position differs in different cell types and this is mediated by cytoskeletal interactions [72]. Much effort has gone into identifying the proteins connecting the nucleus to the cytoskeleton. A combination of techniques identified the ‘linker of nucleoskeleton and cytoskeleton’ (LINC) complex [9,73]. Major components of the LINC complex are members of the Nesprin family of proteins [74]. They are transmembrane proteins that span the outer nuclear membrane binding to the SUN proteins in the intermembrane space between the outer and inner nuclear membranes. The SUN proteins span the inner nuclear membrane and bind to the nuclear lamins, as well as to other proteins such as emerin. Extending into the cytoplasm, the nesprins bind directly or indirectly with the actin, microtubule and intermediate filament cytoskeletons [74]. Exerting tension on isolated nuclei by pulling on nesprins induced a stiffening of the nucleus, confirming that the nucleus is mechanosensitive and will respond to tension transmitted through the cytoskeleton and the LINC complex [75].

Tension exerted on the nucleus is known to affect transcription and the differentiated phenotype of cells. This is an exciting area but for space reasons we will leave this topic to other reviews [76–79] and papers in this volume. Here we will discuss how the nucleus, as a relatively rigid intracellular organelle that is physically connected to the cytoskeleton, affects mechanotransduction and aspects of cell behaviour, such as cell migration.

## 6. Severing the LINC complex

Two major strategies have been used to disconnect the nucleus from the cytoskeleton: complete removal of the nucleus (enucleation) and severing the LINC complex, by expression of dominant negative KASH domains, dominant negative SUN proteins, or by depletion of LINC complex components [73,80,81]. Expression of dominant negative KASH domains that compete with Nesprin binding to the SUN proteins was found to alter the mechanical properties of the cells [80,81]. Rheological assays revealed decreased stiffness of the transfected cell's cytoplasm [81], as well as altered force transmission across the cell and decreased nuclear deformation in response to force applied at the cell surface [80]. Changes were detected in the perinuclear organization of actin stress fibres. Additionally, perturbing the LINC complex in this way also decreased the velocity of cell migration, as well as its directional persistence. This latter effect may reflect altered cell polarity due to disruption of the centrosomal/nuclear axis which was observed in response to expression of dominant negative LINC complex constructs [80]. Dominant negative KASH domains also blocked rearward nuclear movement and reorientation of the centrosome in cells migrating into a scratch wound [82].

The nuclear lamina underlying the inner nuclear membrane is connected to the cytoskeleton via the LINC complex and is responsible for much of the rigidity and shape of the nucleus. Deletion of the Lamin A gene (LMNA−/−) revealed that this Lamin is particularly relevant for these mechanical properties [83–87]. Similar to the effects of expressing dominant negative KASH domains, deletion of the Lamin A gene affects not only nuclear mechanics but also the cytoskeleton, cell polarity and cell migration [85,88]. Expressing Lamin A mutant constructs that correspond to those responsible for the diseases of muscle also recapitulates these effects [85,89,90]. Although changes were not detected in the organization of stress fibres in the Lmna−/− cells, their focal adhesions were smaller [85]. Examining the level of RhoA activity, Hale and colleagues made the striking observation that this was significantly lower in the Lamin A null cells [85]. RhoA activity was also decreased in cells expressing a laminopathic mutant of Lamin A, although in these cells changes in the focal adhesions were not detected. This may reflect that the RhoA activity level was still above a critical threshold.

## 7. Enucleation

Expression of dominant negative LINC complex components or their genetic deletion are precision tools to examine the role of nuclear/cytoskeletal connections. A much cruder approach is to remove the nucleus completely. Although crude, this approach does have the advantage that all connections to the nucleus will have been destroyed, whereas severing the LINC complex may leave other cytoskeletal interactions with the nuclear envelope intact. Strategies for large scale enucleation of cells grown in culture were developed in the early 1970s [91]. The enucleated cells, known as cytoplasts, were observed to contain multiple organelles and to survive for hours or days, depending on the cell type.

When techniques for enucleation were developed there was little, if any, interest in mechanotransduction. However, investigators were interested in whether the nucleus had any effect on a cell's migratory behaviour. Goldman and colleagues found that cytoplasts could migrate on glass coverslips, thereby establishing that possession of a nucleus is not a prerequisite for cell migration [92]. This result was supported by several subsequent studies investigating the migratory properties of cell fragments lacking nuclei. For example, growth cones no longer connected to their nerve cell bodies continued to migrate in culture [93]. Similarly, very small membrane-bound fragments of fibroblast cytoplasm (microplasts) exhibited various motile activities, such as ruffling membranes, filopodial extension and retraction, and membrane blebbing [94]. Some of the fastest migrating cells are fish keratocytes, which display a broad leading lamella. Lamellar fragments lacking a nucleus can exhibit a polarized cytoskeletal organization and directed migration [95]. Studying this phenomenon further, it was found that such fragments assume either a non-polarized symmetrical discoid shape or a polarized organization. In the non-polarized morphology state, they were non-migratory, whereas in the polarized state they exhibited sustained directed migration [96]. Notably, directed migration could be induced in the non-polarized fragments by applying mechanical force to one side.

**Figure 4.**
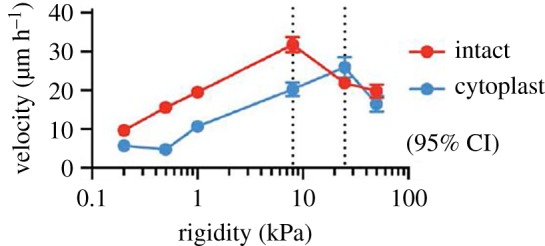
Comparison of migration velocities of intact cells and cytoplasts on substrata of different stiffness. Intact REF52 fibroblasts or REF52 cells that had been enucleated to generate cytoplasts were plated on substrata of different rigidity. Cells were plated on polyacrylamide hydrogels of different compliance (Matrigen™) coated with 10 µg ml^−1^ fibronectin. After allowing cells and cytoplasts to spread for 3 h, individual cells were tracked using an Olympus VivaView microscope and the velocities calculated. Reproduced from Graham *et al.* [99], originally published in the Journal of Cell Biology, doi:10.1083/jcb.201706097. (Online version in colour.)

These earlier studies revealed that many different cell types can migrate efficiently without a nucleus. However, they did not determine the possible role of the nucleus in cells migrating in three dimensions or uncover the extent to which the nucleus contributes to cellular mechanotransduction. Previous work has implicated the nucleus as a major impediment to three-dimensional migration [97]. However, exploring cell migration in three-dimensional cell-derived matrices, Petrie and colleagues found that the nucleus was required for lobopodial migration. They showed that the nucleus acts as a piston, being pulled forward by myosin contractility to generate a pressure differential driving extension of the lobopodial protrusions at the cell front [98]. These opposing earlier conclusions about the role of the nucleus in cell migration stimulated us to use enucleation to explore how the loss of the nucleus affects different types of migration. Deriving cytoplasts from either fibroblasts or endothelial cells, we confirmed that they can migrate on two-dimensional surfaces [99]. Additionally, it was shown that cytoplasts can detect gradients of growth factors and ECM, moving up these by chemotaxis and haptotaxis, respectively. The cytoplasts were also able to close a scratch wound made in a monolayer, although with slightly less efficiency than their intact parent cells. Exploring migration in three-dimensional collagen gels, contrary to expectations, cytoplasts displayed very little net migration. Nevertheless, the cytoplasts were able to extend protrusions into the surrounding three-dimensional matrix but this did not lead to translocation [99]. Why can cells lacking a nucleus migrate so well on two-dimensional surfaces but be so restricted in three-dimensional collagen matrices? Besides the difference in dimensionality, another difference experimentally is that migration on two-dimensional surfaces is usually examined on very stiff substrata (glass or plastic), whereas migration in three-dimensional matrices involves comparatively soft substrata. The stiffness of a three-dimensional matrix is difficult to alter experimentally without simultaneously changing the porosity and/or the ligand density (both factors that will influence migration). However, it is possible to vary the rigidity of two-dimensional surfaces relatively easily. Comparing the migration velocity of cytoplasts and intact cells on surfaces of different rigidity revealed similar velocity profiles but that with cytoplasts equivalent velocities occurred on stiffer substrata (figure 4). Notably, on very soft substrata where the intact cells were still migrating, cytoplasts showed greatly reduced migration. From these experiments, we concluded that at least one explanation for the reduced migration of cytoplasts in three dimensions results from a reduced ability to migrate effectively on or in soft substrata [99].

## 8. Cytoplasts reveal reduced mechanotransduction

The effect of enucleation on migration velocity of intact cells was mimicked by inhibiting myosin II activity with the inhibitor blebbistatin [99]. Treatment of intact cells with blebbistatin shifted the migration velocity peak to stiffer substrata suggesting that enucleation might be affecting myosin activity and cell contractility. A similar effect of myosin inhibition enhancing cell migration on soft substrata has been observed previously [100]. This action of blebbistatin on cytoplasts suggests that removal of the nucleus was affecting overall cell contractility and mechanotransduction. Exploring this further, it was seen that cytoplasts were less able to contract collagen gels and exhibited reduced traction force on the underlying substratum compared with intact cells [99]. Cytoplasts also showed decreased stiffening in response to pulling on magnetic beads coated with fibronectin (DM Graham 2017, unpublished results). Together these results have led us to conclude that loss of the nucleus reduces cell contractility and mechanotransduction. Where in the signalling pathway from mechanical tension to increased contractility is the nucleus having its effect?

**Figure 5.**
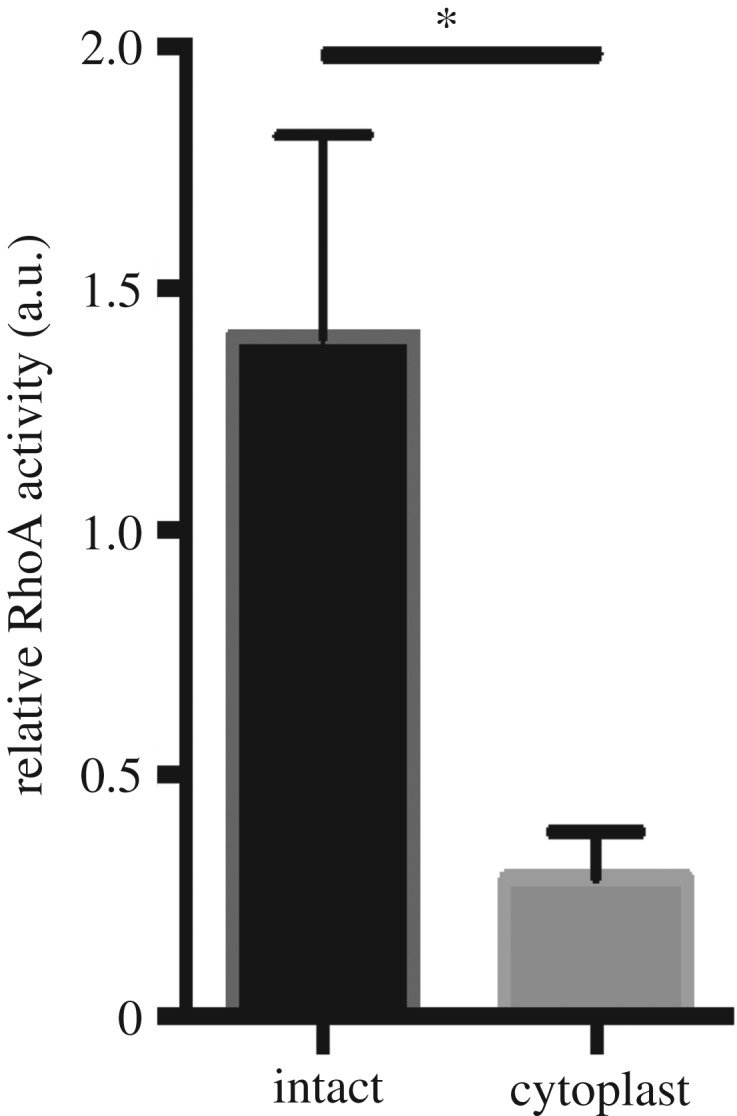
Cytoplasts have decreased RhoA activity compared with intact cells. The levels of active RhoA were measured using the G-Lisa technique (Cytoskeleton™) from parallel cultures of REF52 cells and their corresponding cytoplasts. The experiment was performed in triplicate. Graphical data is represented as a mean with data bars representing s.e.m. *p < 0.05 as determined by *t*-test.

Contractility in nonmuscle cells is the result of myosin II activity, which is regulated by multiple pathways. As mentioned above, a dominant regulatory pathway involves active RhoA stimulating ROCK, which in turn elevates phosphorylation of the regulatory MLC. Comparing the phosphorylation of two ROCK substrates, MYPT and MLC, in cytoplasts with intact cells revealed that both are greatly diminished in cytoplasts (DM Graham 2017, unpublished results). These observations suggest RhoA activity is decreased in cytoplasts, which has been confirmed in direct assays (figure 5).

What is the cause of decreased RhoA activity in cytoplasts? This must reflect either decreased GEF or increased GAP activity. As yet, this has not been resolved. One possibility is that removal of the nucleus depletes certain GEFs such as ECT-2 and NET-1, which are concentrated in the nucleus. How much these GEFs contribute to the overall level of RhoA activity in cells has not been determined. Another possibility is that a rigid nucleus attached to the actin cytoskeleton contributes significantly to the overall tension developed within the cell. Since tension elevates the activities of several GEFs and reciprocally decreases GAP activity (see above), decreasing the tension by removal of the nucleus will be predicted to reduce RhoA activity in this model. This is supported by the observation that cells lacking Lamin A have a soft nucleus, less tension and lower RhoA activity [85]. Similarly, disconnecting the nucleus from the cytoskeleton by severing the LINC complex has been reported to decrease levels of active RhoA [101]. Since tension elevates the activity of GEFs, such as LARG and GEF-H1, it will be important to determine if the activity of these is decreased in cytoplasts. It will also be important to examine whether there is an increase in the activity of any of the GAPs, particularly examining DLC1 and p190RhoGAP.

## 9. Concluding remarks

Mechanical tension exerted on cells typically signals via cell adhesion molecules to increase RhoA activity. So far most attention has been directed towards the activation of specific GEFs, but the level of active RhoA reflects the balance between GEF activation and GAP inhibition. Recent work reveals that the inhibition of GAPs may be as important as activation of GEFs in a cell's response to mechanical tension. We suspect that in most situations both sides of the GEF/GAP equation contribute to controlling the level of active RhoA. Moving forward, we expect more attention will be focused on RhoA GAPs, particularly on DLC1 and p190RhoGAP, both of which have been implicated in the response of cells to mechanical forces. In this review, we have also discussed the role of the nucleus as a physical structure. Severing the LINC complex, or complete removal of the nucleus, alter a cell's mechanotransduction. Much of the change may be due to reduced RhoA activity. The reason why RhoA activity is decreased has not been resolved but evidence supports a model in which a nucleus, physically connected to the cytoskeleton, contributes to the overall level of tension generated within the cytoskeleton. In turn, the level of tension exerted on a cell's adhesions has a critical influence on the level of RhoA activity. A common theme emerges that RhoA signalling is a key regulator of mechanotransduction regardless of whether the mechanical stimulus is internal and derived, for example, from perturbations of the LINC complex, or external, as occurs in cells responding to substrate stiffness in fibrosis.

## Data accessibility

Data in figure 5 was generated by David Graham. His data can be accessed by contacting him at Grahamdavem@Gmail.com
